# Application of antibody phage display to identify potential antigenic neural precursor cell proteins

**DOI:** 10.1186/s40709-020-00123-4

**Published:** 2020-08-02

**Authors:** Ioannis Paspaltsis, Evangelia Kesidou, Olga Touloumi, Roza Lagoudaki, Marina Boziki, Martina Samiotaki, Dimitra Dafou, Theodoros Sklaviadis, Nikolaos Grigoriadis

**Affiliations:** 1 Neurodegenerative Diseases Research Group, Department of Pharmacy, School of Health Sciences, Aristotle University of Thessaloniki, 54124 Thessaloniki, Greece; 2 B’ Department of Neurology, AHEPA General University Hospital of Thessaloniki, Aristotle University of Thessaloniki, 54636 Thessaloniki, Greece; 3 Laboratory of Physiology, Medical School, Aristotle University of Thessaloniki, 54636 Thessaloniki, Greece; 4 Biomedical Sciences Research Center‘Alexander Fleming’, Fleming 34, 16672 Vari, Greece; 5 Department of Genetics, Development, and Molecular Biology, School of Biology, Aristotle University of Thessaloniki, 54124 Thessaloniki, Greece

**Keywords:** Neural precursor cells, NPCs, Recombinant Fab fragments, Antibody phage display

## Abstract

**Background:**

The discovery of neural precursor cells (NPCs) and the concomitant intensive research in the field offer regenerative medicine novel approaches, enabling it to tackle conditions, such as neurodegenerative diseases. Transplantation of NPCs is nowadays considered a cutting-edge treatment for these conditions and many related clinical trials have been already completed or are still ongoing. However, little is known about the antigenicity of NPCs, with most studies addressing the question whether their antigenicity could lead to rejection of the transplanted cells.

**Results:**

In this study we investigated the antigenic potential of syngeneic NPCs emulsion, upon subcutaneous (s.c.) administration to wild type C57BL/6 mice, following a standard immunization protocol. The whole IgG repertoire expressed upon immunization was cloned into a Fab phage display vector. From the created phage display library, Fab expressing clones interacting with NPCs lysate proteins were selected with the biopanning technique. The IgG Fab fragment from clone 65 proved to be reactive against antigens originating from NPCs lysates and/or whole brain lysate in diverse immunological assays.

**Conclusions:**

Using a standard immunization protocol to administer NPCs antigens, and applying the Fab fragment phage display technique, we were able to isolate at least a monoclonal IgG Fab fragment, which interacts with different mouse brain proteins. It is not clear whether such antibodies are produced in the host organisms, following NPCs transplantation.

## Background

Neural precursor cells (NPCs) are multipotent, self-renewing, ectoderm-derived progenitor cells, which support brain tissue homeostasis. Depending on the needs in health or disease, NPCs may differentiate into neurons, astrocytes and oligodendrocytes or they can release soluble factors, which regulate brain functions. In the mammalian brain, NPCs are found in niches mainly located in the dentate gyrus of the hippocampus, the lateral subventricular zone (SVZ) and the olfactory bulb [1, 2].

Isolated NPCs can be cultured in vitro as single cell suspensions. In the presence of EGF and/or FGF2 these suspensions grow into neurospheres. Clustered single cells forming a neurosphere may in turn be enzymatically dissociated and either recultured to produce new neurospheres or used for experimental and therapeutic purposes [2, 3]. As reported, human spinal cord-derived NPCs could retain their initial multipotency even after 18 such passages and could be successfully transplanted into immunodeficient rats [4].

Transplantation of NPCs is considered a promising therapeutic intervention against disorders affecting the central nervous system (CNS), including neurodegenerative diseases. In fact, many clinical trials assessing the therapeutic potential of NPCs transplantation are still ongoing or have been completed [3, 5]. It has been suggested that the therapeutic effects observed after NPCs transplantation occur as a result of their chemotactic migration towards the lesion and in dependence on the disease-produced specific cytokines [6].

The origin of the graft and its immunogenic potential need to be carefully considered when selecting the most suitable graft for transplantation. In this way, adverse reactions are minimized and graft rejection is avoided. In this regard, autologous or syngeneic grafts are considered as safer, since they are rarely rejected, while transplantation of allogeneic grafts is usually associated with higher rejection rates of the donor material [7, 8]. Hence, research on the immunogenicity of transplanted NPCs is primarily focused on whether immune system activation may affect the survival of the transplanted cells and aims at developing strategies to increase the percentage of surviving NPCs following transplantation. On the other hand, the potential of transplanted syngeneic NPCs to trigger humoral immune responses of the host organism remains, to our knowledge, is barely investigated.

In the current study we immunized C57BL/6 mice with syngeneic neurosphere-derived NPCs and cloned the whole IgG repertoire in a Fab phage display library. Phage clones displaying monoclonal Fab fragments on their surface were isolated based on their immunoreactivity against the whole NPCs proteome. Fab sequences were analyzed, recombinant monoclonal Fab fragments were expressed in *Escherichia coli* cells and their reactivity to NPCs lysates was tested. One of the selected clones (clone 65) was able to immunoprecipitate diverse antigens derived from a mouse whole brain lysate. These data suggest that syngeneic NPCs may trigger immune responses resulting in antibody production. Further studies are required to determine whether such antibodies are produced following NPCs transplantation and to delineate the effects of produced antibodies in the context of disease conditions, where NPCs transplantation is performed.

## Results

### Immunization

**Fig. 1:**
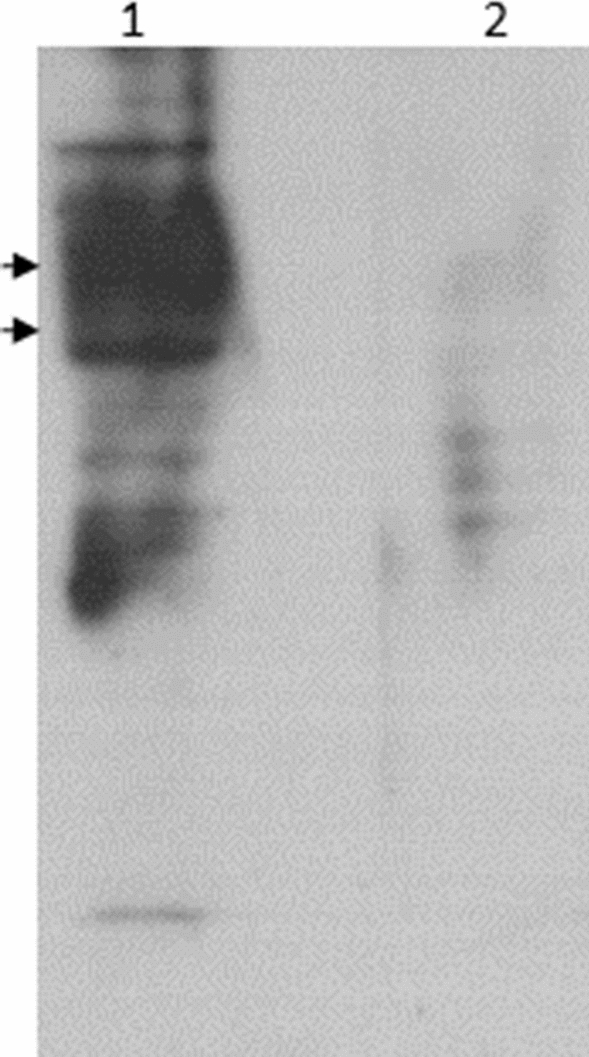
Proteins contained in 20 μg of a NPCs lysate were separated in a 12% polyacrylamide gel and transferred onto PVDF membrane. Individual strips were probed with lane 1: immunized serum 2 and lane 2: the pre immune serum at a 1:1000 dilution in blocking buffer. A HRP-conjugated anti mouse IgG antibody (Cell Signaling, 7076) diluted 1:2000 in blocking buffer, served as secondary antibody. Blot was visualized with the ECL reagent on a autoradiography film. Arrows indicate the positions of the 58 and 80 kDa molecular mass standards

Αntisera harvested from the two immunized mice (serum 1 and 2) were tested for immunoreactivity against proteins from NPCs lysates by immunoblotting. When equally diluted (1:1000), serum 2 displayed stronger reactivity, compared to serum 1. Figure 1 shows NPCs-associated protein bands, recognized by serum 2. The pre-immune serum, in comparison, displays markedly reduced immunoreactivity.

### Library complexity—clone enrichment by biopanning

*Escherichia coli XL1*-*Blue* cells were transformed with the phagemid containing DNA coding for the Fab fragments and the transformants were titrated to estimate the size of the library. Titration results showed that the created unamplified phage library consisted of 2.5 × 10^6^ plaque forming units (pfu). Titration of input and output phage at each biopanning round are shown on Table 1.

**Table 1:** Titration results after each biopanning round

Panning round	Phage input (pfu)^a^	Phage output (pfu)^b^	% Bound^c^	Enrichment factor^d^
1	9.5 × 10^11^	1.20 × 10^6^	1.20 × 10^−6^	–
2	7.3 × 10^11^	0.80 × 10^6^	1.00 × 10^−6^	0.83
3	2.0 × 10^11^	8.48 × 10^7^	4.24 × 10^−4^	424

^a^Number of phage pfu incubated with the antigens immobilized on the nitrocellulose strip

^b^Number of phage pfu eluted after the incubation above

^c^Percentage of bound phage calculated as the ratio phage output/phage input × 100

^d^Ratio of the percentage of bound phage at round n vs the percentage of bound phage at round n–1

### Sequencing results

**Fig. 2:**
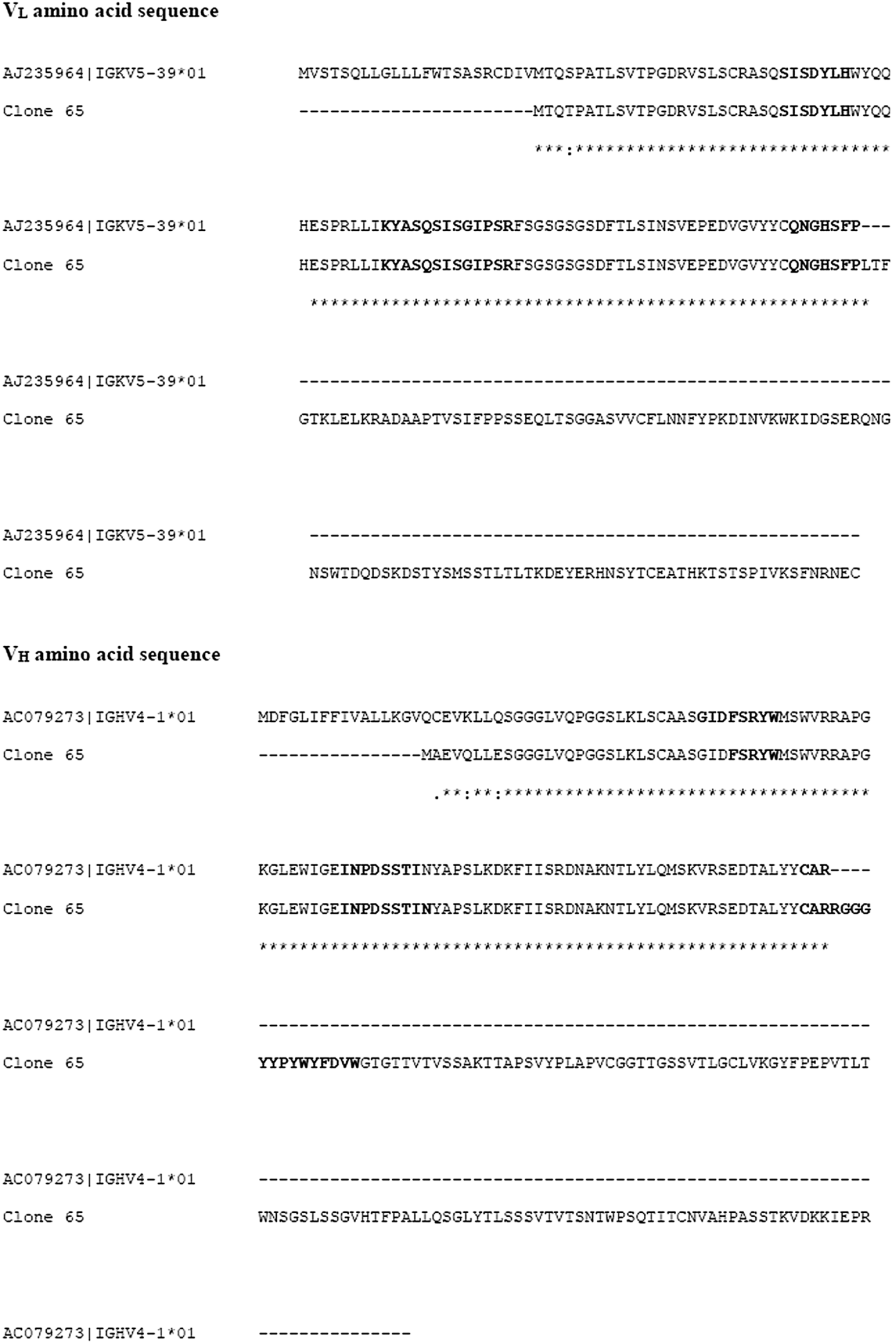
Amino acid sequences of the variable heavy and the light chains from Fab clone 65 compared to the closest germline (IGHV4-01*01 and IGKV5-39*01) sequences as calculated by the IgBLAST software. CDR’s are indicated in bold. *indicates identity to the uppermost sequence, - no amino acid at this position, : indicates the existence of different amino acids at this position

**Fig. 3:**
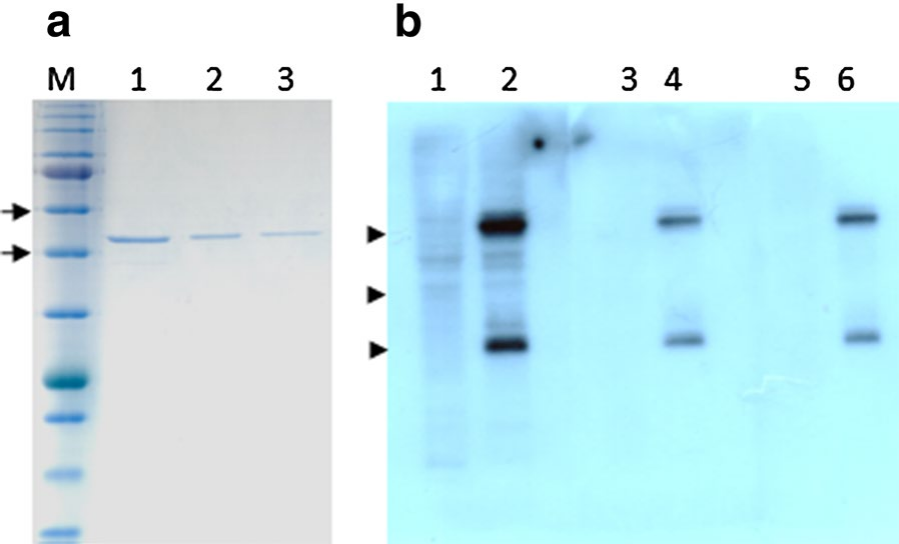
Purification and reactivity of recombinant Fab fragment from clone 65. **a** Purification of Fab 65 from *E. coli Top10F’* crude extracts after IMAC purification and subsequent affinity purification with protein L agarose beads. Samples were separated under non-reducing conditions in a 10% polyacrylamide gel stained with coomassie brilliand blue. M: prestained protein standards (NEB, P7712). Lanes 1–3: three eluates of the last purification step from the protein L agarose beads. Arrows indicate the 46 and 58 kDa molecular mass standard positions, respectively. **b** Western blot. Lanes 1–2: probed with a Fab 65 *E. coli Top10F’* crude extract, lanes 3–4: probed with a *E. coli Top10F’* Fab TT crude extract (kindly provided by the Carlos F. Barbas III laboratory, the Scripps Research Institute), lanes 5–6: probed only with the secondary antibody. Lanes 1, 3, 4: contain 20 μg NPCs lysate, lanes 2, 4, 6: contain 20 μg mouse spinal cord lysate. Arrowheads indicate the 25, 32 and 46 kDa molecular mass standard positions, respectively

More than 20 individual Fab-expressing clones were isolated and the DNA encoding for the Fab was sequenced. Although no clone predomination was observed, two clones were isolated twice. Among the isolated clones, we focused on the recombinant Fab fragment from clone 65 because: (i) we were able to produce it in ample amounts in *E. coli* cells, (ii) it was reactive against NPCs-derived antigens in western blot (Fig. 3b). The primary amino acid sequences of Fab 65 containing the complementarity determining regions (CDRs) from both heavy and light chain are shown in Fig. 2 . Of note, the variable heavy chain domain (V_H_) of clone 65 appeared in four additional Fab clones, combined with different light chains.

### Fab expression and immunoblot reactivity

The recombinant Fab fragments were expressed in *E. coli Top10F*’ cells. The yield varied greatly among the different clones, with some clones producing extremely low amounts of pure Fab’s, while others, including clone 65, generating adequate for further analysis amounts. Figure 3a shows a representative picture of purified Fab 65. Following SDS-PAGE electrophoresis, samples from three elution fractions were stained with coomassie brilliant blue. Electrophoresis was carried out under non reducing conditions, i.e. in the absence of β-mercaptoethanol in the sample buffer. Under these conditions, the expected relative molecular mass of the Fab fragment is about 50 kDa. In the presence of β-mercaptoethanol, the assembled heavy and light chains of the Fab fragment, are resolved into two bands of about 32 and 25 kDa, respectively. The yield of Fab 65 purification under the conditions described in the methods section was approximately 1.0 mg l^−1^ of bacterial culture.

Reactivity of crude extracts from Fab fragment preparations or of purified Fab fragments was assessed by immunoblotting. Both preparations gave similar results, however signals acquired when crude extracts were used were more intense. Fab 65 was shown to recognize not one distinct but several antigens in protein lysates from mouse NPCs or CNS (Fig. 3b, lanes 1–2). Control strips were probed with a human Fab TT, which recognizes the unrelated tetanus toxin (provided by the Carlos F. Barbas III laboratory [9], Fig. 3b, lanes 3–4), or only with the secondary anti-mouse antibody (Fig. 3b, lanes 5–6). The intense bands at 25 and 50 kDa in lanes 2, 4 and 6 correspond to the endogenous light and heavy chains from immunoglobulins of the spinal cord tissue.

### Immunohistochemistry–Immunofluorescence

To further characterize immunoreactivity of the generated Fab fragments, we used them as primary antibodies in immunohistochemical (IHC) staining of mouse brain and spinal cord sections. To confirm signal-specificity, we used control sections in which only the secondary antibody was added. Cells expressing antigens recognized by the Fab’s are stained brown, whereas cell nuclei are counterstained blue by hematoxylin staining. In agreement with the immunoblotting experiments, the monoclonal Fab fragment from clone 65 produced stronger and more specific signals, compared to other isolated Fab’s.

**Fig. 4:**
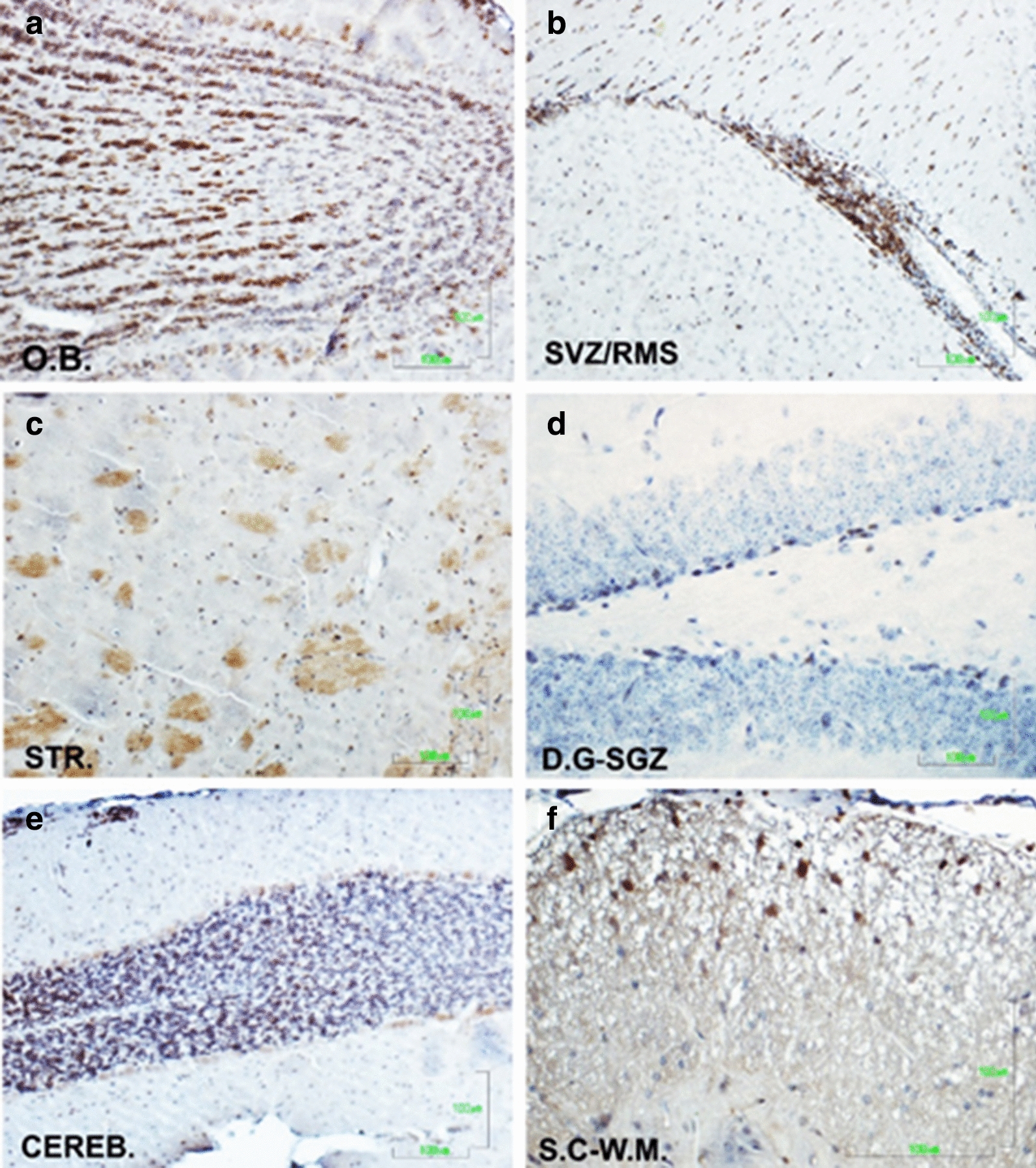
Immunohistochemistry of different mouse brain and spinal cord regions using Fab 65 crude extract as primary antibody. Cells expressing antigens recognized by Fab 65 are stained brown. **a** O.B.: olfactory bulb, **b** SVZ/RMS: subventricular zone/rostral migratory stream of the SVZ, **c** STR.: striatum, **d** D.G-SGZ: subgranular zone of the dentate gyrus, **e** CEREB.: cerebellum, **f** S.C-W.M.: white matter of the spinal cord. Scale bars = 100 μm

Positive reacting cells were identified in all brain regions tested, namely the olfactory bulb, the subventricular zone with the rostral migratory stream, the striatum, the subgranular zone of the dentate gyrus, the cerebellum, as well as the white matter of the spinal cord (Fig. 4).

**Fig. 5:**
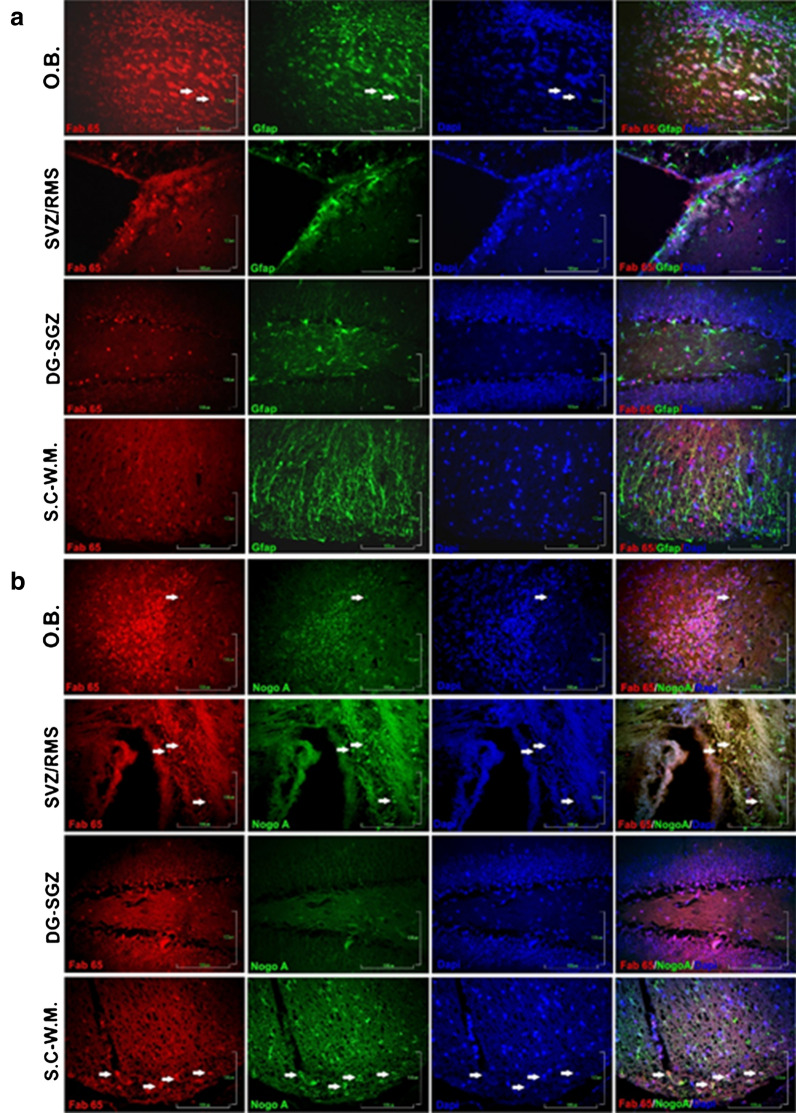
Double immunofluorescence of different mouse CNS regions probed with recombinant Fab65 and a commercial **a** GFAP or **b** Nogo A antibody. O.B.: olfactory bulb, SVZ/RMS: rostral migratory stream of the SVZ, DG-SGZ: subgranular zone of the dentate gyrus, and S.C-W.M.: white matter of the spinal cord. Sections probed with Fab 65 (red) and anti-GFAP or anti-NOGO A (green). Arrows indicate representative cells with observed co-expression of the Fab 65 recognised antigen(s) with GFAP. Cell nuclei were stained with DAPI. Scale bars = 100 μm

Double immunofluorescence (dIF) was conducted in order to determine the cellular localization of the IHC-positive cells. Thus, mouse brain sections were probed with both Fab 65 crude extracts and either a commercial GFAP or a Nogo A antibody. As shown in Fig. 5, signal colocalization between Fab 65 and the anti-GFAP antibody occurred only in astrocytes of the olfactory bulb at a ratio of ~ 17%. No colocalization was observed in the other brain regions tested (the rostral migratory stream of the SVZ, the subgranular zone of the dentate gyrus, and the white matter of the spinal cord). When sections were double-stained with Fab 65 and the anti-Nogo A antibody, double staining was more prominent in oligodendrocytes (Fig. 5). Results from the dIFs are summarized in Additional file 1. Interestingly, there is no relevant signal colocalization observed on cells in the subgranular zone of the dentate gyrus.

### Immunoprecipitation–mass spectrometry

**Fig. 6:**
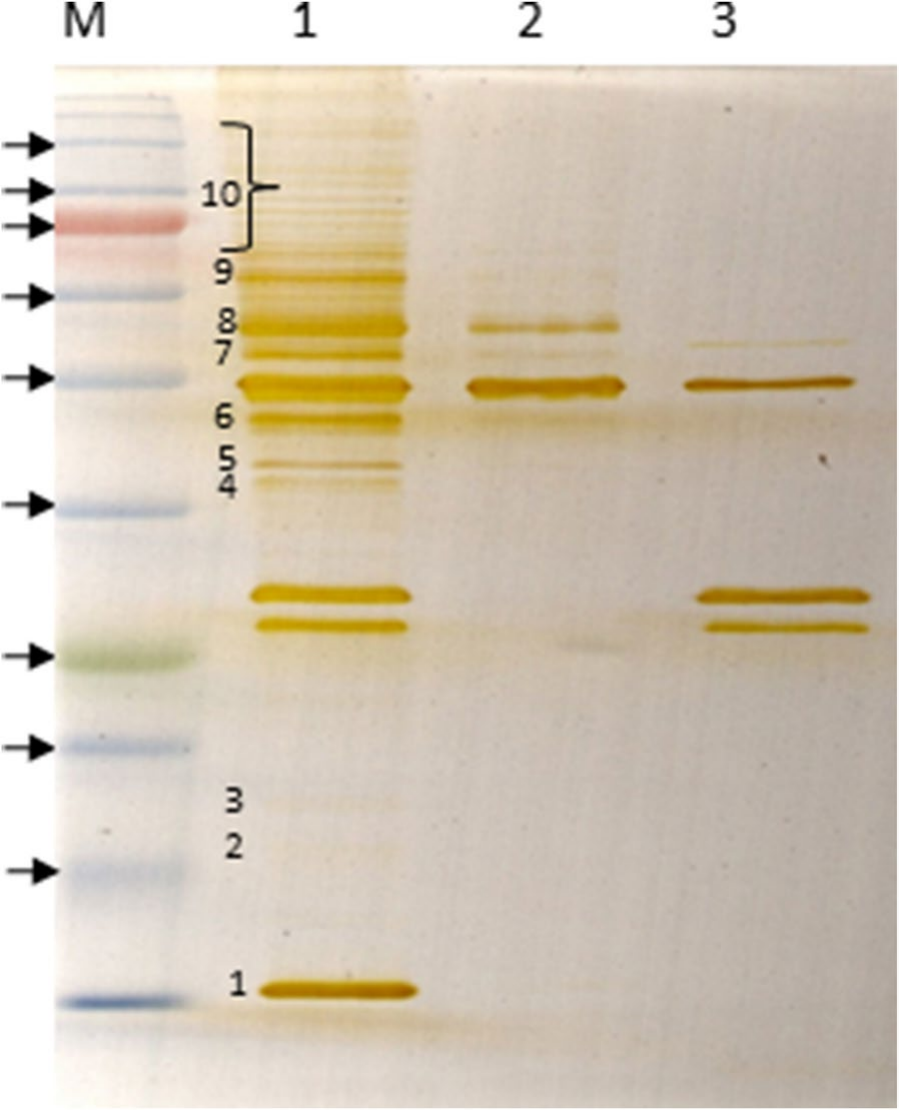
Immunoprecipitation of total mouse brain proteins with Fab 65. 12% polyacrylamide gel was stained with silver ions. Lane 1: 15 μl of the immunoprecipitation reaction in the presence of mouse brain cell lysate and recombinant Fab 65 immobilized on protein L agarose beads. Lane 2: 15 μl of the pre-clearing reaction derived after incubation of the protein L agarose beads with the lysate, Lane 3: 15 μl of Fab 65 immobilized on protein L agarose. Numbers 1–10 on lane 1 indicate the excised protein bands used for mass spectrometry. Spot number 10 was considered as a single band. Arrows indicate the 17, 22, 25, 32, 46, 58, 80, 100 and 135 kDa molecular mass standard positions

Fab 65 immunoreactivity in total mouse brain lysate was further estimated in immunoprecipitation experiments (Fig. 6). In this case, mouse brain lysates were incubated with Fab 65, immobilized on protein L agarose beads or control beads. Immunoprecipitated proteins were resolved by SDS-PAGE and silver-stained. Figure 6 shows that a variety of brain proteins were detected by silver stain after incubation of brain lysate with protein L agarose immobilized Fab 65 (lane 1). These proteins are absent in the control reactions run in parallel, which consisted of protein L agarose beads incubated with the lysate in the absence of the Fab fragment (lane 2) or of the Fab fragment bound protein L agarose beads in the absence of the protein lysate (lane 3). Since SDS-PAGE was performed under reducing conditions, the two distinct protein bands slightly above the 25 kDa marker in lanes 1 and 3 correspond to the light (expected molecular mass: 25 kDa) and heavy (expected molecular mass: 32 kDa) chain of the Fab fragment. In addition, the protein band detected in all three lanes corresponds to protein L (36 kDa), which was released of the agarose beads after heating. Results from the immunoprecipitation experiments with Fab 65 indicate that the Fab fragment recognizes multiple antigens rather than one in a total mouse brain lysate.

To identify the proteins recognized by Fab 65, we carried out analysis of the protein bands indicated in Fig. 6 by mass spectrometry. This analysis revealed a vast variety of proteins, which precipitated in the presence of Fab 65 (Additional file 2). We assume that, some of these are pooled down non-specifically, since they are probably detected due to their binding to other proteins having affinity to Fab 65.

## Discussion

In the present study we applied the antibody fragment phage display technique for the isolation of reactive monoclonal Fab fragments not to a distinct protein but to potential antigenic proteins existing in the whole NPCs proteome. To achieve that, the Fab phage library was created upon immunization of mice with a syngeneic C57/BL6 lysate, derived from a high number (2 × 10^6^ cells) of NPCs, along with immunization adjuvants. This approach certainly does not simulate the administration of NPCs as transplants for therapeutic purposes, since the presence of CFA in the initial immunization and of IFA in the ensuing boosters, as well as the boosters themselves enhance the humoral response. However, this approach aims at and eventually succeeds in the isolation of monoclonal antibodies with affinity to certain NPCs or brain-derived proteins, thus allowing to investigate the generation of such antibodies after a “regular” NPC transplantation into the mouse CNS as well as the effects these may have.

A common problem associated with transplantations is the low survival rate of neural progenitors after engraftment [10]. Antigens from cells that do not survive may trigger immune responses, hence the CNS is no more considered as an immunologically privileged site. In fact, there are indications that repeated CNS allografts may evoke immunization reactions [11]. Moreover, it has been shown that humoral response from immunized animals with myelin oligodendrocyte glycoprotein—MOG_35–55_ produces antibodies that may target and affect NPCs of the SVZ niche, cells that are the orchestrators of neuronal remyelination [12].

The immunized phage library that was created consists of 2.5 × 10^6^ cfu and is considered to be moderate, since more but even less complex libraries have been reported. As a measure, an immunized phage display library contains 99% of all antibody molecules when 1 × 10^7^ individual clones are represented [13, 14]. Since the phagemids of the created library are stored, a freshly prepared library could be easily reamplified on demand. This would enable us to further screen for the existence of IgG Fab fragments recognizing distinct NPCs antigens should this be considered relevant in the future.

Expression of recombinant Fab fragments in the periplasm of *E. coli* cells using shake flasks often leads to relatively low yields, depending on several factors [15]. In fact, most Fab clones isolated in the current study displayed poor yields. However, clone 65 was expressed in satisfactory amounts, with a yield of about 1.0 mg l^−1^ of culture. Expressed Fab was used in immunoblotting and IHC experiments preferably as crude extract as previously described [16] in order to minimize activity reduction occurring during handling for purification purposes. In these experiments, Fab 65 performed better than the other clones, in terms of recognizing NPCs or CNS antigens.

In immunoblotting and immunoprecipitation experiments, Fab 65 was shown to recognize not only one specific but rather multiple proteins, a characteristic of low affinity polyreactive antibodies [17]. This is supported also by the 100% homology observed in its primary structure, compared to the closest germline antibody (Fig. 2). Such antibodies might have an advantage in the biopanning procedure, since the Fab phage library interacts with the multiple proteins present in the NPCs lysate.

IHC experiments showed that Fab 65 is able to bind to cell antigens in the different mouse brain regions tested. Other Fab clones isolated were able to react with cell antigens in some of the brain regions tested, while others showed no reactivity at all. For example, Fab from clone 17 reacted with antigens on neuronal axons, cells in the subventricular zone and the white matter of the spinal cord but no signal was detected on parenchymal cells of the brain. Furhter, Fab’s from clones 68 and 49 showed no reactivity in none of the tested brain regions. As shown in Additional file 1, dIF experiments revealed that Fab 65 binds mainly to antigens on cells expressing Nogo A, a specific oligodendrocyte marker, and the highest co-localization signal, ~ 50%, was observed in the white matter of the spinal cord.

Immunoprecipitation and mass spectrometry analyses did not indicate any clear Fab 65 binding candidate(s). Interestingly, the highest scoring protein in the intense band 1 immunoprecipitated by Fab 65 (Fig. 6) was identified by mass spectrometry to be β-globin. It remains unclear whether this finding should be attributed to the β-globin, present in dopaminergic neurons, astrocytes and oligodendrocytes [18] or to tissue blood contamination.

Due to the high number of proteins identified by Fab 65, binding specificity is difficult to estimate. However, we are in the process of transforming the Fab fragment into full IgG antibody and express it in eukaryotic cell lines. We anticipate that the full IgG glycosylated antibody will be generated with much improved yields and most importantly it will have enhanced affinity and avidity, due to the bivalency and the presence of the Fc region [19].

## Conclusions

By creating an immunized Fab fragment phage display library, we were able to isolate more than 20 monoclonal Fab’s reacting with NPCs antigens. One clone, clone 65, was expressed as recombinant Fab in *E. coli* cells in adequate amounts. More importantly, this clone was reactive against NPCs or other mouse brain cell antigens in different immunological tests. Our data show that Fab 65 does recognize multiple antigens rather than one specific protein. The reactivity of the other clones requires more detailed investigation. Whether such antibodies might be generated during NPCs transplantations for therapeutic purposes and the effects these antibodies may have will be investigated in future experiments.

## Methods

### Neurospheres preparation

Neurospheres were prepared from NPCs derived from brain hemispheres of newborn C57BL/6 mice as previously described [12]. Briefly, isolated NPCs from the brain hemispheres were initially treated with trypsin, then mechanically dissociated and afterwards resuspended in DMEM/F2 media containing the N-2 supplement (Invitrogen, Carlsbad, USA). Cells were cultured for 7 days at 37 °C, 10% CO_2_ in the presence of 10 μg ml^−1^ basic fibroblast growth factor (bFGF) and 10 μg ml^−1^ epidermal growth factor (EGF). Developed neurospheres were harvested by centrifugation. About 10^6^ cells were resuspended for protein extraction in 100 μl lysis buffer containing 25 mM Tris pH 7.5, 150 mM NaCl, 1 mM DTT, 1 mM PMSF, 1% Triton X-100, 12 μg ml^−1^ leupeptin and 5 μg ml^−1^ aprotinin. Incubation for 15 min was followed by centrifugation for 10 min at 10,000×*g* at room temperature to remove cell debris. The resulting supernatant was aliquoted and stored at − 80 °C for future use. Protein content was estimated by the Bradford method.

### Immunization

Two 6-week-old C57BL/6 male mice were immunized subcutaneously with 200 μl of protein lysate derived from 2 × 10^6^ NPCs in sterile phosphate buffered saline (PBS) pH 7.4. Cells were stored frozen at − 80 °C. Before immunization cells were emulsified in complete Freund’s adjuvant (CFA) in a ratio of 1:1. Booster immunizations with the same dosage were performed similarly 2, 4 and 8 weeks after the initial immunization, using incomplete Freund’s adjuvant (IFA). Spleens were harvested and processed for RNA extraction. Blood was collected via submandibular bleeding before and after immunization to obtain preimmune and immune sera.

### RNA extraction and amplification of Fab fragment sequences

Total RNA was extracted using the Trizol reagent (Invitrogen, Carlsbad, USA) following the manufacturer’s instructions. Tissue was homogenized using the Ribolyzer apparatus with 1 μm ceramic beads for three cycles of 20 s each (at setting 4). In a second purification step, mRNA was extracted from the total RNA generated in the previous step, using the poly A Spin mRNA isolation kit (NEB, Ipswich, USA) following the manufacturer’s instructions. Isolated mRNA was quantified with the NanoDrop apparatus (Thermo Fisher Scientific, Waltham, USA).

10 μg of mRNA were subjected to cDNA synthesis with the iScript cDNA synthesis kit (Biorad, Hercules, USA). cDNAs were pooled and used as template for PCR amplification of the IgG Fab fragments i.e. the heavy chain variable domains (V_H_) and the constant framework region 1 (C_H1_) along with the κ light chains (variable V_κ_ and constant C_κ_ domains), with the PCR primers given in Additional file 3. PCR was performed with Q5 high fidelity DNA polymerase (NEB, Ipswich, USA). For heavy chain (HC) fragment amplification primer pairs of each primer 1–7 as forward and each primer 8–11 as reverse were used. Similarly, primer combinations for light chain amplification included primer 19 as reverse primer and one of the primers 12–18 as forward. After an initial denaturation step at 94 °C for 5 min, HC fragment cyclic amplification conditions were: 94 °C for 20 s, 68 °C for 20 s, 72 °C for 1 min (35 cycles) and finally 72 °C for 7 min. The same conditions were applied for LCs amplification, except for the annealing temperature, which was set at 56 °C. The products from all HC PCRs were pooled together, as were the products from the LC PCRs and then resolved in a 1.8% TAE agarose gel. The ~ 680 bp products were gel-excised and purified with the Nucleospin Gel and PCR Clean Up kit (Macherey–Nagel, Düren, Germany) according to the manufacturer’s instructions. Purified products were stored at − 20 °C until needed.

### Cloning into pComb3XSS vector

pComb3XSS vector was kindly provided by the Carlos F. Barbas III laboratory (Scripps Research Institute). Cloning of the Fab fragment sequences was performed as described previously [20]. In more detail, 2 μg of the vector and approximately 400 ng of PCR amplified LCs were mixed in separate reactions with restriction enzymes *Sac*I (TaKaRa, Kyoto, Japan) 20 U and *Xba*I (TaKaRa, Kyoto, Japan) 30 U in 1× buffer M and incubated overnight at 37 °C, followed by heat inactivation for 10 min at 65 °C. Reaction products were separated in a 1.2% TAE agarose gel and both the restriction-digested vector and the DNA coding for the LCs were gel-excised and purified as above. Purified products were quantified in Nanodrop apparatus and LC sequences were ligated to the corresponding *Sac*I/*Xba*I sites of the vector. Ligation products were purified by sodium acetate precipitation and electroporated into electrocompetent *E. coli XL*-*1 Blue* cells, using the MicroPulser apparatus (Biorad, Hercules, USA). A small aliquot of the transformed *E. coli* cells was used for titration of the transformants; the remaining cells were incubated overnight at 37 °C in 500 ml Super Broth (SB, 30 g l^−1^ tryptone, 20 g l^−1^ yeast extract, 10 g l^−1^ MOPS pH 7.0) in the presence of 100 μg ml^−1^ carbenicillin and 15 μg ml^−1^ tetracycline. The vector containing the LCs sequences was purified using the maxi prep kit (Macherey–Nagel, Düren, Germany) and used for the cloning of the HC fragments. To this end, 1 μg of the PCR-amplified HC fragments and 5 μg of the pComb-LC library derived in the previous step, were digested overnight at 37 °C with 60 U of the enzymes *Spe*I HF and *Xho*I (both from NEB, Ipswich, USA) in 1× cut smart buffer. After a heat inactivation step at 80 °C for 10 min, digestion products were separated in a 1.2% agarose gel in TAE, gel-excised and purified as before. Purified products were quantified in Nanodrop apparatus and HC sequences were ligated to the corresponding *Spe*I/*Xho*I sites of the vector. Ligation products were purified by precipitation with sodium acetate and electroporated into electrocompetent *E. coli XL*-*1 Blue* cells. Titration of the derived transformants was performed by plating serial dilutions of a small aliquot on LB plates (10 g l^−1^ tryptone, 5 g l^−1^ yeast extract, 5 g l^−1^ NaCl, 17 g l^−1^ agar, pH 7.0) containing 50 μg ml^−1^ carbenicillin and 15 μg ml^−1^ tetracycline. Carbenicillin 100 μg ml^−1^ and tetracycline 15 μg ml^−1^ were added to the transformed cells SB and incubated for 2 h at 37 °C with vigorous shaking before the addition of 2 × 10^11^ pfu of the helper phage M13KO7 (NEB, Ipswich, USA), which was followed by further overnight incubation under the same conditions. Bacteria cells were pelleted by centrifugation at 3000×*g* for 15 min at 4 °C and processed for phagemid purification. Phages were purified from the supernatant by 4% w/v PEG 8000, 3% w/v NaCl precipitation for 30 min at 4 °C. The final phage pellet, corresponding to the Fab phage display library, was resuspended in sterile TBS (50 mM Tris pH 7.5, 150 mM NaCl) containing 1% bovine serum albumin (BSA) and stored at 4 °C.

### Biopanning of the Fab library on NPCs protein lysates

Phage clones from the immune library displaying IgG antibody Fab fragments with affinity to NPCs proteins were selected and enriched by the biopanning procedure [20]. NPCs proteins were immobilized on nitrocellulose membrane, an approach reported to be efficient in similar experiments [21]. 20 μg of a NPCs lysate were separated by SDS-PAGE on a 12% polyacrylamide gel and electrotransferred as described in the immunoblotting section. Nitrocellulose membrane was cut in strips and non-specific binding sites were blocked with PBS containing 2% BSA. Furthermore, pure nitrocellulose strips with no protein bound were similarly blocked. These served as a substrate on which 50 μl (about 10^11^ pfu) of the Fab phage library diluted in 450 μl PBS, 0.1% v/v Tween 20 were incubated for 30 min at room temperature to remove nitrocellulose and/or BSA binding clones from the supernatant. Last was removed and incubated with the NPCs proteins immobilized on a nitrocellulose strip for 2 h at room temperature. The strip was washed 5× with 5 ml PBST. Bound phage clones were eluted by incubation of the strip for 10 min at room temperature with a low pH buffer (100 mM glycine pH 2.2). The eluate was neutralized with 2 M Tris base. Serial dilutions of an aliquot were used for the titration of the phages in the eluate by infecting an *E. coli XL*-*1 Blue* culture and plating on LB plates containing 50 μg ml^−1^ carbenicillin and 15 μg ml^−1^ tetracycline. An *E. coli XL*-*1 Blue* culture in SB media at OD_600_ = 0.8–1.0 was infected with the eluate in the presence of the above antibiotics and super infected with the M13 helper phage as previously described [20]. Propagated phage was purified, titered as before and served as the input phage for the second round of biopanning. In total, three biopanning rounds were performed. To enhance stringency of the selection conditions, PBS 0.5% v/v Tween 20 was used by washing in round two and three. Also, washing step number was increased to 10 and 15 in round two and three, respectively. After completion of round three, phage infected *E. coli XL*-*1 Blue* clones on LB plates containing 50 μg ml^−1^ carbenicillin and 15 μg ml^−1^ tetracycline were picked for phagemid isolation with the Nucleospin plasmid kit (Macherey–Nagel, Düren, Germany). Chemically competent *E. coli Top10F’* cells were transformed with aliquots of the phagemid. Transformants were plated on LB plates containing 50 μg ml^−1^ carbenicillin and incubated overnight at 37 °C. Single colonies were picked up and cultured in SB, 100 μg ml^−1^ carbenicillin for plasmid isolation, which was further used for DNA sequencing or for the expression of recombinant Fab fragments.

### Sequence analysis

DNA sequencing of the plasmids was performed at CeMIA SA (Greece) by the Sanger method using primers 20–21 (Additional file 3) as forward and reverse primer, respectively. Nucleic acid sequences were analyzed with the IMGT/V-QUEST software [22] while the corresponding amino acid variable domains were analyzed with IgBLAST [23] in order to identify the germlines and to locate the complementarity determining regions (CDRs) of the IgG Fab fragments expressing clones.

### Expression of recombinant Fab fragments

Monoclonal recombinant IgG Fab fragments were expressed in *E. coli Top10F*’ cells. A liquid culture in SB, 100 μg ml^−1^ carbenicillin, 1% w/v glucose (sterile filtrated) was set up by picking a colony from freshly transformed cells. After overnight incubation at 37 °C with shaking, the culture was diluted 1:100 in 500 μl SB, 100 μg ml^−1^ carbenicillin, 0.1% w/v glucose and incubated as before until OD_600_ = 0.8 was reached. At that point, MgCl_2_ (20 mM final concentration) was added, while Fab expression was induced by the addition of 0.5 mM isopropyl-d-thiogalactopyranoside (IPTG). Culture was incubated for 24 h at 25 °C with shaking. Bacteria cells were pelleted by centrifugation at 3000×*g*, 4 °C for 30 min. Cells were resuspended in 20 ml PBS and treated with 20 mg ml^−1^ lysozyme for 30 min at room temperature. Further, cell suspension underwent three freeze-thaw cycles. Released bacterial DNA was digested by DNase I (0.5 mg ml^−1^ for 15 min at room temperature). Cell-debris was removed by centrifugation (10,000×*g* for 15 min at 4 °C). The supernatant, containing Fab fragments, was aliquoted and stored at − 20 °C. This crude extract was either used directly for antigen detection by immunoblot/IHC or for Fab fragment purification. Soluble Fabs were first purified over the His-tag of the Fab heavy chain by Ni-chelate affinity chromatography with Protino Ni–NTA agarose (Macherey–Nagel, Düren, Germany) following the manufacturer’s instructions. Eluates were diluted 1:1 with PBS and mixed with protein L agarose beads (ABT). After incubation for 1 h under rotation at room temperature, samples were centrifuged at 100×*g*, 5 min at room temperature. Pellet was washed 2× with 1 ml PBS and the purified Fab finally eluted with 0.1 M citric acid pH 2.8. pH of the eluates was neutralized immediately with 1 M Tris pH 9.0.

### SDS-PAGE electrophoresis and immunoblotting

SDS-PAGE electrophoresis was conducted according to the Leammli method [24] on 10 or 12% polyacrylamide gels. For immuno-detection, separated proteins were transferred onto PVDF membranes, blocked with blocking buffer (PBS, 0.1% v/v Tween 20, 5% w/v non fat dried milk) for 1 h at room temperature. Blots were incubated with Fab preparations diluted 1:1 in blocking buffer as specified for additional 2 h. After 4× wash steps in PBS 0.1% Tween 20 for 10 min, membranes were incubated with a HRP-conjugated anti mouse antibody diluted in blocking buffer and washed as before. Reactivity was visualized with the ECL reagent. Where necessary, separated proteins were stained non-specifically with coomassie brilliant blue or for enhanced sensitivity with silver ions.

### Immunohistochemistry–immunofluorescence

Immunohistochemistry and immunofluorescence experiments were performed on naive mouse C57BL/6 paraffin brain sections as described in [12]. As primary antibodies, *E. coli Top10F*’ crude extract preparations were used, diluted 1:5 in PBS containing 10% FBS. The reactivity assessment of the different Fab clones was visualized by developing with DAB under an optical microscope. dIF experiments were performed for the co-expression estimation of Fab 65 reactive antigens with Nogo A and/or GFAP proteins, which served as oligodendrocytes and astrocytes markers, respectively. Both marker molecules were detected with commercial anti-rabbit antibodies (Santa Cruz Biotechnology, Santa Cruz, USA). 4′,6-diamidino-2-phenylindole (DAPI) staining was followed in order to visualize cell nuclei. Fluorescence microscope images were analyzed using the ImageJ 1.52 b software [25].

### Immunoprecipitation

The Fab fragment from clone 65 was first purified by Ni-chelate affinity chromatography using 0.5 ml of a Fab 65 *E. coli Top10F*’ crude extract preparation. The 250 mM imidazole-containing eluate was diluted 1:1 with PBS and incubated with 50 μl of protein L agarose beads in order to remove impurities copurified on the Ni–NTA column. Affinity purification was performed as described above. After the last centrifugation step, instead of eluting, beads were incubated with 1 ml of a precleared mouse brain homogenate in RIPA buffer (150 mM NaCl, 1.0% v/v IGEPAL CA-630, 0.5% w/v sodium deoxycholate, 0.1% w/v SDS, 50 mM Tris, pH 8.0) containing about 3 mg of total protein. Incubation was performed for 3 h at room temperature on a test tube rotator. The mix was centrifuged at 100×*g*, 5 min at room temperature. The pellet containing the Fab 65 bound protein L agarose beads and possible pulled down proteins was washed 3× with 1 ml PBS. After the last centrifugation step, the supernatant was aspirated and 60 μl of 2× sample buffer were added. For preclearing, mouse brain homogenate was incubated with 50 μl of protein L agarose beads for 1 h as described above. Protein L agarose beads were handled as the immunoprecipitation sample, as an assessment for mouse brain proteins with affinity to the agarose beads. A negative control reaction consisted of Fab 65 bound protein L agarose in the absence of any brain protein lysate. In all cases, proteins were heated at 95 °C for 5 min, separated into a 12% polyacryamide gel and stained subsequently with silver ions as described [26].

### In-gel digestion of proteins with trypsin

In gel protein digestion was performed according to standard procedures [27]. Briefly, the gel pieces were destained, and subjected to two cycles of dehydration with acetonitrile (ACN) and rehydration with 25 mM NH_4_HCO_3_ buffer. Followed dehydration with ACN, the dried gel pieces were rehydrated with 12.5 ng µl^−1^ trypsin (Trypsingold, Promega, Madison, USA) in 25 mM NH_4_HCO_3_ buffer and incubated over night at 37 °C. Peptides were extracted from the gel with 100 μl extraction buffer [1:2 (v/v) 5% formic acid/ACN] for 30 min at 37 °C and the solution was transferred and dried in a vacuum centrifuge. Finally, the samples were reconstituted in 2% v/v ACN/0.1% v/v formic acid and sonicated in a water bath for 5 min.

### LC–MS/MS analysis

The purified peptides were analyzed by HPLC-tandem MS/MS using a C-18 column coupled to a LTQ Orbitrap XL Mass spectrometer (Thermo Scientific, Waltham, USA). 10 μl of peptides were pre-concentrated at a flow of 5 μl min^−1^ for 10 min using a C18 trap column (Acclaim PepMap RSLC, Thermo Scientific, Waltham, USA) and then loaded onto a 15 cm C18 column (75 μm ID, particle size 2 μm, 100 Å, Acclaim PepMap RSLC, Thermo Scientific, Waltham, USA). The binary pumps of the HPLC (RSLC nano, Thermo Scientific, Waltham, USA) contained solution A [2% (v/v) ACN in 0.1% (v/v) formic acid] and solution B (80% ACN in 0.1% formic acid). The peptides were separated using a linear gradient of 4–40% B in 60 min at a flow rate of 300 nl min^−1^. The column was placed in an oven operating at 35 °C. Full scan MS spectra were acquired in the orbitrap (m/z 300–1600) in profile mode and data-dependent acquisition with the resolution set to 60,000 at m/z 400 and automatic gain control target at 106. The six most intense ions were sequentially isolated for collision-induced MS/MS fragmentation (normalized CID of 35%) and detection in the linear ion trap. Dynamic exclusion was set to 60 s. Ions with single charge states were excluded. Lockmass of m/z 445,120,025 was used for internal calibration. The software Xcalibur (Thermo Scientific, Waltham, USA) was used to control the system and acquire the raw files.

### Data analysis and database search

Peptides were identified using the Proteome Discoverer 1.4 software tools (Thermo Scientific, Waltham, USA). The Orbitrap raw data (peak S/N threshold was set to 1.5) were searched using SEQUEST HT against the uniprot mouse FASTA database with strict trypsin specificity and with maximum two missed cleavages and variable modifications of methionine oxidation and acetylation of the N-terminus. The peptides were filtered based on their Xcorr values versus peptide charge states (XCorr > 2.0 for charge state + 2 and XCorr > 2.5 for charge state + 3).

## Supplementary information

**Additional file 1.** Plot illustrating double immunofluorescence experiment results concerning co-expression of the Fab 65 recognised antigen(s) with GFAP or NOGO**. A.** Co-expression with GFAP is observed only in the olfactory bulb (O.B) at about 17%. Co-expression with NOGO A is observed in the olfactory bulb, in the subventricular zone/rostral migratory stream of the SVZ (SVZ/RMS) and in white matter of the spinal cord (SC/WM) at about 33%, 51 and 58% respectively. No co-expression with neither of the glial marker antigens was detected in the subgranular zone of the dentate gyrus (DG/SGZ). Statistical analysis was performed with the GraphPad Prism 5.0 software using the Shapiro–Wilk and Kolmogorov–Smirnov tests. Values were analyzed by the ANOVA test followed by the Bonferoni post hoc test. Graph shows mean values and the standard error of the mean out of three different regions of the sections tested. Bars indicate the standard error of the mean of three different section regions analyzed.

**Additional file 2.** Mass spectrometric analysis results of Fab 65 immunoprecipitated brain protein bands. Immunoprecipitated proteins from a whole C57/BL6 mouse brain lysate using recombinant Fab 65 were electrophoretically separated onto a 12% polyacrylamide gel. Protein bands were excised as they are numbered on Fig. 6 and analyzed by mass spectrometry. As “Protein Band 10” is referred the whole region indicated on the figure. The proteins listed presended the highest counts regarding the parameter of unique peptides. Numbering is the same as on Fig. 6.

**Additional file 3.** PCR primers used in this study. Primers 1–19 were used for the amplification of IgG Fab fragments, while primers 20–21 for the sequencing of the isolated phagemids.Degenerative nucleotide symbols:K = G or T, S = C or G, M = A or C, W = A or T, R = A or G, Y = C or T. Restriction enzymes recognition sites are underlined, with A|CTAGT for *Spe*I, C|TCGAG for *Xho*I, T|CTAGA for *Xba*I and GAGCT|C for *Sac*I.

## Data Availability

Please contact author for data requests.

## Abbreviations

bFGF: Basic fibroblast growth factor
CFA: Complete Freund’s adjuvant
cfu: Colony forming units
CNS: Central nervous system
DAB: 3,3′ Diaminobenzidine-tetra-hydrochloride
DAPI: 4′,6-Diamidino-2-phenylindole
ECL: Enhanced chemiluminescence
EGF: Epidermal growth factor
GFAP: Glial fibrillary acidic protein
HRP: Horse radish peroxidase
IFA: Incomplete Freund’s adjuvant
NPCs: Neural precursor cells
PBS: Phosphate buffered saline
pfu: Plaque forming units
SGZ: Subgranular zone
SVZ: Subventricular zone
