# Supplementary material for: Application of antibody phage display to identify potential antigenic neural precursor cell proteins
Source: J Biol Res (Thessalon). 2020 Aug 2;27:14. doi: 10.1186/s40709-020-00123-4 (PMC7398072; doi:10.1186/s40709-020-00123-4)
Supplement: Supplementary file 1 — Additional file 1. Plot illustrating double immunofluorescence experiment results concerning co-expression of the Fab 65 recognised antigen(s) with GFAP or NOGO. A. Co-expression with GFAP is observed only in the olfactory bulb (O.B) at about 17%. Co-expression with NOGO A is observed in the olfactory bulb, in the subventricular zone/rostral migratory stream of the SVZ (SVZ/RMS) and in white matter of the spinal cord (SC/WM) at about 33%, 51 and 58% respectively. No co-expression with neither of the glial marker antigens was detected in the subgranular zone of the dentate gyrus (DG/SGZ). Statistical analysis was performed with the GraphPad Prism 5.0 software using the Shapiro–Wilk and Kolmogorov–Smirnov tests. Values were analyzed by the ANOVA test followed by the Bonferoni post hoc test. Graph shows mean values and the standard error of the mean out of three different regions of the sections tested. Bars indicate the standard error of the mean of three different section regions analyzed. [file 40709_2020_123_MOESM1_ESM.docx]

**Additional file 1**

**Plot illustrating double immunofluorescence experiment results concerning co-expression of the Fab 65 recognised antigen(s) with GFAP or NOGO A.**


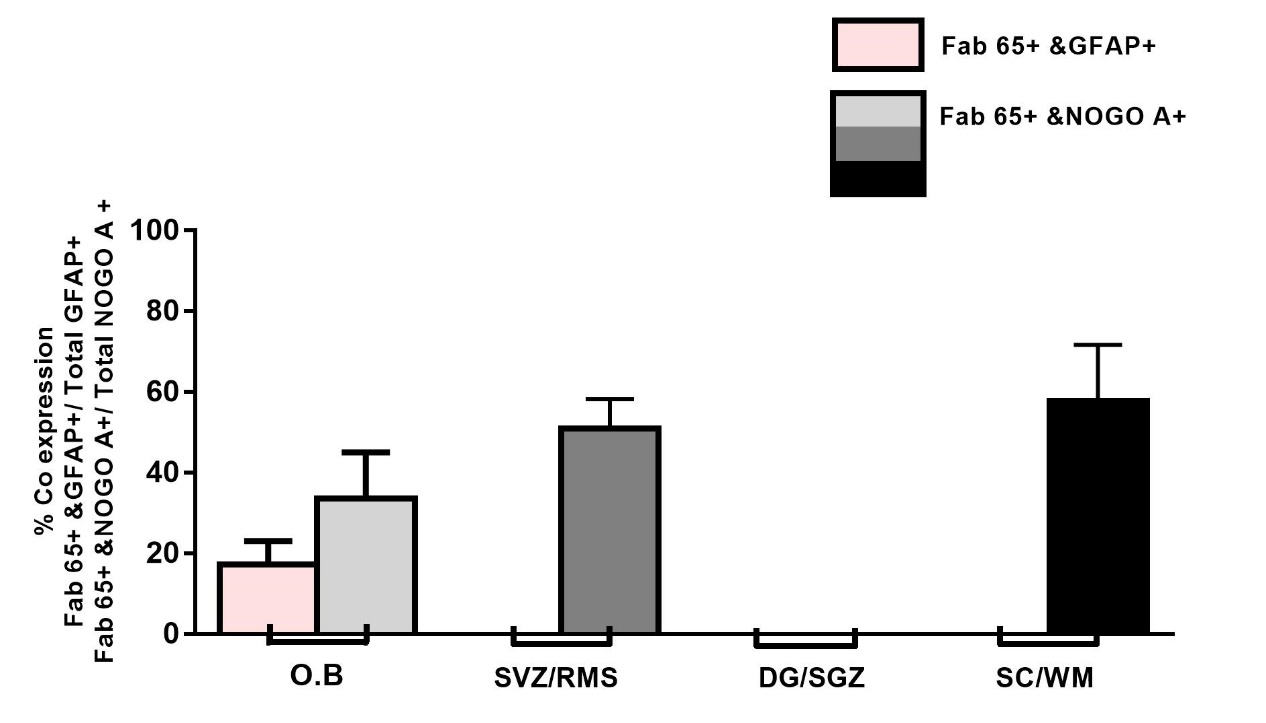


Co-expression with GFAP is observed only in the olfactory bulb (O.B) at about 17%. Co-expression with NOGO A is observed in the olfactory bulb, in the subventricular zone/ rostral migratory stream of the SVZ (SVZ/RMS) and in white matter of the spinal cord (SC/WM) at about 33%, 51 and 58% respectively. No co-expression with neither of the glial marker antigens was detected in the subgranular zone of the dentate gyrus (DG/SGZ). Statistical analysis was performed with the GraphPad Prism 5.0 software using the Shapiro-Wilk and Kolmogorov-Smirnov tests. Values were analyzed by the ANOVA test followed by the Bonferoni post-hoc test. Graph shows mean values and the standard error of the mean out of three different regions of the sections tested.Bars indicate the standard error of the mean of three different section regions analyzed.
