# Supplementary material for: Application of antibody phage display to identify potential antigenic neural precursor cell proteins
Source: J Biol Res (Thessalon). 2020 Aug 2;27:14. doi: 10.1186/s40709-020-00123-4 (PMC7398072; doi:10.1186/s40709-020-00123-4)
Supplement: Supplementary file 2 — Additional file 2. Mass spectrometric analysis results of Fab 65 immunoprecipitated brain protein bands. Immunoprecipitated proteins from a whole C57/BL6 mouse brain lysate using recombinant Fab 65 were electrophoretically separated onto a 12% polyacrylamide gel. Protein bands were excised as they are numbered on Fig. 6 and analyzed by mass spectrometry. As “Protein Band 10” is referred the whole region indicated on the figure. The proteins listed presended the highest counts regarding the parameter of unique peptides. Numbering is the same as on Fig. 6. [file 40709_2020_123_MOESM2_ESM.docx]

**Additional file 2**

**Mass spectrometry analysis results of Fab 65 immunoprecipitated brain protein bands as they are numbered on Figure 4.**

**Protein Band 1**

| Accession | Description | Score | Coverage | # Proteins | # Unique Peptides | # Peptides | # PSMs | # AAs | MW [kDa] | calc. pI |
| --- | --- | --- | --- | --- | --- | --- | --- | --- | --- | --- |
| A8DUK4 | Beta-globin OS=Mus musculus GN=Hbbt1 PE=1 SV=1 - [A8DUK4_MOUSE] | 102.36 | 58.50 | 3 | 5 | 7 | 30 | 147 | 15.7 | 7.69 |
| P01942 | Hemoglobin subunit alpha OS=Mus musculus GN=Hba PE=1 SV=2 - [HBA_MOUSE] | 30.98 | 30.28 | 2 | 3 | 3 | 9 | 142 | 15.1 | 8.22 |
| P26883 | Peptidyl-prolyl cis-trans isomerase FKBP1A OS=Mus musculus GN=Fkbp1a PE=1 SV=2 - [FKB1A_MOUSE] | 22.00 | 29.63 | 2 | 3 | 3 | 6 | 108 | 11.9 | 8.16 |
| P12787 | Cytochrome c oxidase subunit 5A, mitochondrial OS=Mus musculus GN=Cox5a PE=1 SV=2 - [COX5A_MOUSE] | 21.82 | 17.12 | 1 | 3 | 3 | 7 | 146 | 16.1 | 6.54 |

**Protein Band 2**

| Accession | Description | Score | Coverage | # Proteins | | # Unique Peptides | | # Peptides | # PSMs | | | # AAs | | MW [kDa] | | calc. pI |
| --- | --- | --- | --- | --- | --- | --- | --- | --- | --- | --- | --- | --- | --- | --- | --- | --- |
| P17742 | Peptidyl-prolyl cis-trans isomerase A OS=Mus musculus GN=Ppia PE=1 SV=2 - [PPIA_MOUSE] | 30.10 | 46.95 | 4 | 5 | | 5 | | | 9 | 164 | | 18.0 | | 7.90 | |
| P02535-3 | Isoform 3 of Keratin, type I cytoskeletal 10 OS=Mus musculus GN=Krt10 - [K1C10_MOUSE] | 20.47 | 9.74 | 6 | 4 | | 4 | | | 7 | 462 | | 49.5 | | 5.02 | |
| Q01768 | Nucleoside diphosphate kinase B OS=Mus musculus GN=Nme2 PE=1 SV=1 - [NDKB_MOUSE] | 19.39 | 34.21 | 5 | 4 | | 4 | | | 6 | 152 | | 17.4 | | 7.50 | |
| P62761 | Visinin-like protein 1 OS=Mus musculus GN=Vsnl1 PE=1 SV=2 - [VISL1_MOUSE] | 14.34 | 26.18 | 1 | 4 | | 4 | | | 4 | 191 | | 22.1 | | 5.15 | |

**Protein Band 3**

| Accession | Description | Score | Coverage | # Proteins | # Unique Peptides | # Peptides | # PSMs | # AAs | MW [kDa] | calc. pI | |
| --- | --- | --- | --- | --- | --- | --- | --- | --- | --- | --- | --- |
| P18760 | Cofilin-1 OS=Mus musculus GN=Cfl1 PE=1 SV=3 - [COF1_MOUSE] | 33.55 | 42.77 | 2 | 5 | 6 | 9 | 166 | 18.5 | | 8.09 |
| P04370-5 | Isoform 5 of Myelin basic protein OS=Mus musculus GN=Mbp - [MBP_MOUSE] | 30.14 | 36.69 | 18 | 5 | 5 | 10 | 169 | 18.5 | | 11.15 |
| P02535-3 | Isoform 3 of Keratin, type I cytoskeletal 10 OS=Mus musculus GN=Krt10 - [K1C10_MOUSE] | 30.20 | 11.69 | 7 | 4 | 5 | 11 | 462 | 49.5 | | 5.02 |
| Q9D6J5 | NADH dehydrogenase [ubiquinone] 1 beta subcomplex subunit 8, mitochondrial OS=Mus musculus GN=Ndufb8 PE=1 SV=1 - [NDUB8_MOUSE] | 18.39 | 35.48 | 5 | 4 | 4 | 6 | 186 | 21.9 | | 6.64 |
| Q4VAE3 | Transmembrane protein 65 OS=Mus musculus GN=Tmem65 PE=1 SV=1 - [TMM65_MOUSE] | 17.12 | 14.96 | 1 | 4 | 4 | 6 | 234 | 24.9 | | 7.78 |
| Q9DCJ5 | NADH dehydrogenase [ubiquinone] 1 alpha subcomplex subunit 8 OS=Mus musculus GN=Ndufa8 PE=1 SV=3 - [NDUA8_MOUSE] | 16.79 | 28.49 | 1 | 4 | 4 | 6 | 172 | 20.0 | | 8.46 |
| P62748 | Hippocalcin-like protein 1 OS=Mus musculus GN=Hpcal1 PE=1 SV=2 - [HPCL1_MOUSE] | 14.44 | 25.91 | 1 | 4 | 4 | 5 | 193 | 22.3 | | 5.50 |
| P84096 | Rho-related GTP-binding protein RhoG OS=Mus musculus GN=Rhog PE=1 SV=1 - [RHOG_MOUSE] | 12.62 | 35.60 | 1 | 4 | 4 | 4 | 191 | 21.3 | | 8.12 |

**Protein Band 4**

| Accession | Description | Score | Coverage | # Proteins | # Unique Peptides | # Peptides | # PSMs | # AAs | MW [kDa] | calc. pI |
| --- | --- | --- | --- | --- | --- | --- | --- | --- | --- | --- |
| P08249 | Malate dehydrogenase, mitochondrial OS=Mus musculus GN=Mdh2 PE=1 SV=3 - [MDHM_MOUSE] | 51.38 | 36.39 | 3 | 10 | 10 | 17 | 338 | 35.6 | 8.68 |
| P61264 | Syntaxin-1B OS=Mus musculus GN=Stx1b PE=1 SV=1 - [STX1B_MOUSE] | 31.61 | 32.99 | 1 | 9 | 9 | 10 | 288 | 33.2 | 5.38 |
| P14152 | Malate dehydrogenase, cytoplasmic OS=Mus musculus GN=Mdh1 PE=1 SV=3 - [MDHC_MOUSE] | 25.70 | 20.36 | 2 | 5 | 5 | 9 | 334 | 36.5 | 6.58 |
| P02535-3 | Isoform 3 of Keratin, type I cytoskeletal 10 OS=Mus musculus GN=Krt10 - [K1C10_MOUSE] | 19.46 | 11.69 | 11 | 4 | 5 | 8 | 462 | 49.5 | 5.02 |
| D6RFB9 | Syntaxin-1A OS=Mus musculus GN=Stx1a PE=1 SV=1 - [D6RFB9_MOUSE] | 19.09 | 22.66 | 3 | 4 | 4 | 6 | 256 | 29.5 | 4.98 |
| O35129 | Prohibitin-2 OS=Mus musculus GN=Phb2 PE=1 SV=1 - [PHB2_MOUSE] | 12.18 | 14.38 | 3 | 4 | 4 | 4 | 299 | 33.3 | 9.83 |

**Protein Band 5**

| Accession | Description | Score | Coverage | # Proteins | # Unique Peptides | # Peptides | # PSMs | # AAs | MW [kDa] | calc. pI |
| --- | --- | --- | --- | --- | --- | --- | --- | --- | --- | --- |
| P08249 | Malate dehydrogenase, mitochondrial OS=Mus musculus GN=Mdh2 PE=1 SV=3 - [MDHM_MOUSE] | 59.41 | 52.66 | 3 | 13 | 14 | 24 | 338 | 35.6 | 8.68 |
| Q9CWS0 | N(G),N(G)-dimethylarginine dimethylaminohydrolase 1 OS=Mus musculus GN=Ddah1 PE=1 SV=3 - [DDAH1_MOUSE] | 36.35 | 50.88 | 2 | 10 | 12 | 16 | 285 | 31.4 | 5.97 |
| P16858 | Glyceraldehyde-3-phosphate dehydrogenase OS=Mus musculus GN=Gapdh PE=1 SV=2 - [G3P_MOUSE] | 81.87 | 42.94 | 8 | 9 | 11 | 57 | 333 | 35.8 | 8.25 |
| Q8K0S0 | Phytanoyl-CoA hydroxylase-interacting protein OS=Mus musculus GN=Phyhip PE=1 SV=1 - [PHYIP_MOUSE] | 39.61 | 33.33 | 2 | 7 | 13 | 21 | 330 | 37.5 | 7.01 |
| P46096 | Synaptotagmin-1 OS=Mus musculus GN=Syt1 PE=1 SV=1 - [SYT1_MOUSE] | 21.2 | 23.75 | 4 | 7 | 11 | 12 | 421 | 47.4 | 8.53 |
| P51863 | V-type proton ATPase subunit d 1 OS=Mus musculus GN=Atp6v0d1 PE=1 SV=2 - [VA0D1_MOUSE] | 25.4 | 25.64 | 1 | 5 | 9 | 15 | 351 | 40.3 | 5.00 |
| O35639 | Annexin A3 OS=Mus musculus GN=Anxa3 PE=1 SV=4 - [ANXA3_MOUSE] | 14.40 | 34.37 | 3 | 5 | 10 | 10 | 323 | 36.4 | 5.76 |
| Q60900-2 | Isoform HuC-S of ELAV-like protein 3 OS=Mus musculus GN=Elavl3 - [ELAV3_MOUSE] | 24.60 | 29.44 | 2 | 4 | 11 | 18 | 360 | 38.8 | 9.28 |
| O70439 | Syntaxin-7 OS=Mus musculus GN=Stx7 PE=1 SV=3 - [STX7_MOUSE] | 10.28 | 24.9 | 2 | 4 | 6 | 6 | 261 | 29.8 | 5.78 |

**Protein Band 6**

| Accession | Description | Score | Coverage | Proteins | Unique Peptides | # Peptides | # PSM | # AAs | MW [kDa] | calc. pI |
| --- | --- | --- | --- | --- | --- | --- | --- | --- | --- | --- |
| Q93092 | Transaldolase OS=Mus musculus GN=Taldo1 PE=1 SV=2 - [TALDO_MOUSE] | 21 | 25.22 | 1 | 8 | 10 | 10 | 337 | 37.36 | 7.03 |
| P05063 | Fructose-bisphosphate aldolase C OS=Mus musculus GN=Aldoc PE=1 SV=4 - [ALDOC_MOUSE] | 83 | 57.58 | 2 | 12 | 21 | 44 | 363 | 39.37 | 7.12 |
| P05202 | Aspartate aminotransferase, mitochondrial OS=Mus musculus GN=Got2 PE=1 SV=1 - [AATM_MOUSE] | 76 | 41.4 | 1 | 11 | 21 | 36 | 430 | 47.38 | 8.99 |
| P63085 | Mitogen-activated protein kinase 1 OS=Mus musculus GN=Mapk1 PE=1 SV=3 - [MK01_MOUSE] | 59 | 41.62 | 23 | 10 | 15 | 26 | 358 | 41.25 | 6.97 |
| P05201 | Aspartate aminotransferase, cytoplasmic OS=Mus musculus GN=Got1 PE=1 SV=3 - [AATC_MOUSE] | 42 | 41.16 | 2 | 10 | 18 | 22 | 413 | 46.22 | 7.13 |
| P05064 | Fructose-bisphosphate aldolase A OS=Mus musculus GN=Aldoa PE=1 SV=2 - [ALDOA_MOUSE] | 92 | 49.18 | 9 | 8 | 15 | 43 | 364 | 39.33 | 8.08 |
| P18872-2 | Isoform Alpha-2 of Guanine nucleotide-binding protein G(o) subunit alpha OS=Mus musculus GN=Gnao1 - [GNAO_MOUSE] | 47 | 41.81 | 17 | 8 | 14 | 20 | 354 | 40.01 | 6.01 |
| Q62420 | Endophilin-A1 OS=Mus musculus GN=Sh3gl2 PE=1 SV=2 - [SH3G2_MOUSE] | 43 | 44.6 | 6 | 6 | 15 | 21 | 352 | 39.93 | 5.38 |
| B2RSH2 | Guanine nucleotide-binding protein G(i) subunit alpha-1 OS=Mus musculus GN=Gnai1 PE=1 SV=1 - [GNAI1_MOUSE] | 31 | 35.31 | 19 | 4 | 11 | 14 | 354 | 40.34 | 5.97 |

**Protein Band 7**

| Accession | Description | Score | Coverage | # Proteins | # Unique Peptides | # Peptides | # PSMs | # AAs | MW [kDa] | calc. pI |
| --- | --- | --- | --- | --- | --- | --- | --- | --- | --- | --- |
| P17182 | Alpha-enolase OS=Mus musculus GN=Eno1 PE=1 SV=3 - [ENOA_MOUSE] | 61.95 | 31.80 | 9 | 8 | 12 | 22 | 434 | 47.1 | 6.80 |
| P17183 | Gamma-enolase OS=Mus musculus GN=Eno2 PE=1 SV=2 - [ENOG_MOUSE] | 43.08 | 31.57 | 8 | 8 | 11 | 13 | 434 | 47.3 | 5.11 |
| Q61598-2 | Isoform 2 of Rab GDP dissociation inhibitor beta OS=Mus musculus GN=Gdi2 - [GDIB_MOUSE] | 35.86 | 33.74 | 2 | 4 | 12 | 15 | 409 | 46.6 | 6.90 |
| Q9R111 | Guanine deaminase OS=Mus musculus GN=Gda PE=1 SV=1 - [GUAD_MOUSE] | 26.87 | 21.59 | 2 | 7 | 9 | 10 | 454 | 51.0 | 5.53 |
| P97807-2 | Isoform Cytoplasmic of Fumarate hydratase, mitochondrial OS=Mus musculus GN=Fh - [FUMH_MOUSE] | 25.42 | 21.20 | 2 | 5 | 7 | 9 | 467 | 50.0 | 7.94 |
| Q9D8N0 | Elongation factor 1-gamma OS=Mus musculus GN=Eef1g PE=1 SV=3 - [EF1G_MOUSE] | 24.20 | 13.04 | 1 | 6 | 7 | 9 | 437 | 50.0 | 6.74 |
| P10630 | Eukaryotic initiation factor 4A-II OS=Mus musculus GN=Eif4a2 PE=1 SV=2 - [IF4A2_MOUSE] | 20.74 | 20.15 | 5 | 5 | 7 | 7 | 407 | 46.4 | 5.48 |
| P10637-4 | Isoform Tau-C of Microtubule-associated protein tau OS=Mus musculus GN=Mapt - [TAU_MOUSE] | 20.57 | 26.10 | 9 | 4 | 6 | 8 | 341 | 35.7 | 9.44 |
| Q9CZ13 | Cytochrome b-c1 complex subunit 1, mitochondrial OS=Mus musculus GN=Uqcrc1 PE=1 SV=2 - [QCR1_MOUSE] | 18.77 | 15.00 | 3 | 5 | 6 | 7 | 480 | 52.8 | 6.21 |
| Q62465 | Synaptic vesicle membrane protein VAT-1 homolog OS=Mus musculus GN=Vat1 PE=1 SV=3 - [VAT1_MOUSE] | 18.68 | 19.21 | 1 | 5 | 6 | 7 | 406 | 43.1 | 6.37 |
| Q99JY9 | Actin-related protein 3 OS=Mus musculus GN=Actr3 PE=1 SV=3 - [ARP3_MOUSE] | 17.40 | 15.07 | 4 | 5 | 5 | 6 | 418 | 47.3 | 5.88 |

**Protein Band 8**

| Accession | Description | Score | Coverage | # Proteins | # Unique Peptides | # Peptides | # PSMs | # AAs | MW [kDa] | calc. pI |
| --- | --- | --- | --- | --- | --- | --- | --- | --- | --- | --- |
| P26443 | Glutamate dehydrogenase 1, mitochondrial OS=Mus musculus GN=Glud1 PE=1 SV=1 - [DHE3_MOUSE] | 77.56 | 30.29 | 2 | 14 | 14 | 24 | 558 | 61.3 | 8.00 |
| P62814 | V-type proton ATPase subunit B, brain isoform OS=Mus musculus GN=Atp6v1b2 PE=1 SV=1 - [VATB2_MOUSE] | 44.23 | 30.92 | 2 | 12 | 12 | 14 | 511 | 56.5 | 5.81 |
| Q03265 | ATP synthase subunit alpha, mitochondrial OS=Mus musculus GN=Atp5a1 PE=1 SV=1 - [ATPA_MOUSE] | 44.55 | 24.59 | 3 | 10 | 10 | 15 | 553 | 59.7 | 9.19 |
| P68369 | Tubulin alpha-1A chain OS=Mus musculus GN=Tuba1a PE=1 SV=1 - [TBA1A_MOUSE] | 96.53 | 46.12 | 5 | 6 | 15 | 29 | 451 | 50.1 | 5.06 |
| Q3UJQ9 | Succinyl-CoA:3-ketoacid-coenzyme A transferase OS=Mus musculus GN=Oxct1 PE=1 SV=1 - [Q3UJQ9_MOUSE] | 32.77 | 17.49 | 2 | 6 | 6 | 10 | 486 | 52.2 | 8.94 |
| Q61644 | Protein kinase C and casein kinase substrate in neurons protein 1 OS=Mus musculus GN=Pacsin1 PE=1 SV=1 - [PACN1_MOUSE] | 20.11 | 19.73 | 1 | 6 | 6 | 6 | 441 | 50.5 | 5.24 |
| Q9CPY7-2 | Isoform 2 of Cytosol aminopeptidase OS=Mus musculus GN=Lap3 - [AMPL_MOUSE] | 17.90 | 17.01 | 3 | 6 | 6 | 6 | 488 | 52.7 | 7.03 |

**Protein Band 9**

| Accession | Description | Score | Coverage | # Proteins | # Unique Peptides | # Peptides | # PSMs | # AAs | MW [kDa] | calc. pI |
| --- | --- | --- | --- | --- | --- | --- | --- | --- | --- | --- |
| O08553 | Dihydropyrimidinase-related protein 2 OS=Mus musculus GN=Dpysl2 PE=1 SV=2 - [DPYL2_MOUSE] | 91.89 | 41.26 | 1 | 15 | 16 | 29 | 572 | 62.2 | 6.38 |
| Q9EQF6 | Dihydropyrimidinase-related protein 5 OS=Mus musculus GN=Dpysl5 PE=1 SV=1 - [DPYL5_MOUSE] | 65.60 | 27.84 | 2 | 12 | 12 | 21 | 564 | 61.5 | 7.09 |
| Q3TT92 | Dihydropyrimidinase-related protein 3 OS=Mus musculus GN=Dpysl3 PE=1 SV=1 - [Q3TT92_MOUSE] | 62.62 | 35.92 | 5 | 12 | 13 | 20 | 568 | 61.7 | 6.49 |
| O35098 | Dihydropyrimidinase-related protein 4 OS=Mus musculus GN=Dpysl4 PE=1 SV=1 - [DPYL4_MOUSE] | 53.61 | 38.11 | 4 | 12 | 14 | 19 | 572 | 61.9 | 6.98 |
| P52480 | Pyruvate kinase PKM OS=Mus musculus GN=Pkm PE=1 SV=4 - [KPYM_MOUSE] | 41.73 | 28.44 | 2 | 10 | 10 | 12 | 531 | 57.8 | 7.47 |
| P63038 | 60 kDa heat shock protein, mitochondrial OS=Mus musculus GN=Hspd1 PE=1 SV=1 - [CH60_MOUSE] | 36.37 | 23.21 | 4 | 9 | 9 | 11 | 573 | 60.9 | 6.18 |
| Q8R081 | Heterogeneous nuclear ribonucleoprotein L OS=Mus musculus GN=Hnrnpl PE=1 SV=2 - [HNRPL_MOUSE] | 31.95 | 30.89 | 4 | 9 | 9 | 9 | 586 | 63.9 | 8.10 |
| O88935-1 | Isoform Ib of Synapsin-1 OS=Mus musculus GN=Syn1 - [SYN1_MOUSE] | 41.78 | 21.04 | 5 | 8 | 8 | 11 | 670 | 70.0 | 9.83 |
| E9Q133 | T-complex protein 1 subunit gamma OS=Mus musculus GN=Cct3 PE=1 SV=1 - [E9Q133_MOUSE] | 23.07 | 17.95 | 4 | 8 | 8 | 8 | 507 | 56.5 | 6.70 |
| P26645 | Myristoylated alanine-rich C-kinase substrate OS=Mus musculus GN=Marcks PE=1 SV=2 - [MARCS_MOUSE] | 28.70 | 41.42 | 1 | 7 | 7 | 9 | 309 | 29.6 | 4.34 |
| H3BL49 | T-complex protein 1 subunit theta OS=Mus musculus GN=Cct8 PE=1 SV=1 - [H3BL49_MOUSE] | 22.01 | 18.40 | 5 | 7 | 7 | 7 | 489 | 53.0 | 5.50 |
| Q9D0F9 | Phosphoglucomutase-1 OS=Mus musculus GN=Pgm1 PE=1 SV=4 - [PGM1_MOUSE] | 26.10 | 13.88 | 2 | 6 | 6 | 9 | 562 | 61.4 | 6.57 |
| P50396 | Rab GDP dissociation inhibitor alpha OS=Mus musculus GN=Gdi1 PE=1 SV=3 - [GDIA_MOUSE] | 19.90 | 19.02 | 3 | 6 | 6 | 6 | 447 | 50.5 | 5.08 |
| P61979-3 | Isoform 3 of Heterogeneous nuclear ribonucleoprotein K OS=Mus musculus GN=Hnrnpk - [HNRPK_MOUSE] | 18.78 | 18.91 | 13 | 6 | 6 | 6 | 439 | 48.5 | 5.92 |
| O08599 | Syntaxin-binding protein 1 OS=Mus musculus GN=Stxbp1 PE=1 SV=2 - [STXB1_MOUSE] | 17.32 | 12.46 | 2 | 6 | 6 | 6 | 594 | 67.5 | 6.96 |

**Protein Band 10**

| Accession | Description | Score | Coverage | # Proteins | # Unique Peptides | # Peptides | # PSMs | # AAs | MW [kDa] | calc. pI |
| --- | --- | --- | --- | --- | --- | --- | --- | --- | --- | --- |
| Q99KI0 | Aconitate hydratase, mitochondrial OS=Mus musculus GN=Aco2 PE=1 SV=1 - [ACON_MOUSE] | 49.30 | 17.56 | 1 | 11 | 13 | 16 | 780 | 85.4 | 7.93 |
| P63017 | Heat shock cognate 71 kDa protein OS=Mus musculus GN=Hspa8 PE=1 SV=1 - [HSP7C_MOUSE] | 70.57 | 27.40 | 7 | 10 | 15 | 26 | 646 | 70.8 | 5.52 |
| Q68FD5 | Clathrin heavy chain 1 OS=Mus musculus GN=Cltc PE=1 SV=3 - [CLH1_MOUSE] | 39.48 | 11.34 | 3 | 10 | 13 | 13 | 1675 | 191.4 | 5.69 |
| O88935-1 | Isoform Ib of Synapsin-1 OS=Mus musculus GN=Syn1 - [SYN1_MOUSE] | 52.58 | 34.48 | 3 | 9 | 11 | 15 | 670 | 70.0 | 9.83 |
| P20029 | 78 kDa glucose-regulated protein OS=Mus musculus GN=Hspa5 PE=1 SV=3 - [GRP78_MOUSE] | 41.52 | 24.43 | 1 | 9 | 11 | 14 | 655 | 72.4 | 5.16 |
| Q02053 | Ubiquitin-like modifier-activating enzyme 1 OS=Mus musculus GN=Uba1 PE=1 SV=1 - [UBA1_MOUSE] | 30.47 | 11.06 | 2 | 8 | 8 | 10 | 1058 | 117.7 | 5.66 |
| Q6PIC6 | Sodium/potassium-transporting ATPase subunit alpha-3 OS=Mus musculus GN=Atp1a3 PE=1 SV=1 - [AT1A3_MOUSE] | 64.25 | 23.79 | 8 | 7 | 19 | 21 | 1013 | 111.6 | 5.41 |
| P38647 | Stress-70 protein, mitochondrial OS=Mus musculus GN=Hspa9 PE=1 SV=3 - [GRP75_MOUSE] | 28.00 | 16.64 | 1 | 7 | 8 | 10 | 679 | 73.4 | 6.07 |
| P46460 | Vesicle-fusing ATPase OS=Mus musculus GN=Nsf PE=1 SV=2 - [NSF_MOUSE] | 23.84 | 13.98 | 2 | 7 | 9 | 9 | 744 | 82.6 | 6.95 |
| P07901 | Heat shock protein HSP 90-alpha OS=Mus musculus GN=Hsp90aa1 PE=1 SV=4 - [HS90A_MOUSE] | 37.10 | 18.28 | 5 | 6 | 12 | 13 | 733 | 84.7 | 5.01 |
| P50516 | V-type proton ATPase catalytic subunit A OS=Mus musculus GN=Atp6v1a PE=1 SV=2 - [VATA_MOUSE] | 30.15 | 17.18 | 5 | 6 | 7 | 11 | 617 | 68.3 | 5.58 |
| P16546-2 | Isoform 2 of Spectrin alpha chain, non-erythrocytic 1 OS=Mus musculus GN=Sptan1 - [SPTN1_MOUSE] | 24.20 | 3.96 | 6 | 6 | 8 | 9 | 2452 | 282.2 | 5.31 |
| O08599 | Syntaxin-binding protein 1 OS=Mus musculus GN=Stxbp1 PE=1 SV=2 - [STXB1_MOUSE] | 23.26 | 15.99 | 2 | 6 | 9 | 9 | 594 | 67.5 | 6.96 |
| Q91VD9 | NADH-ubiquinone oxidoreductase 75 kDa subunit, mitochondrial OS=Mus musculus GN=Ndufs1 PE=1 SV=2 - [NDUS1_MOUSE] | 22.52 | 12.93 | 2 | 6 | 6 | 8 | 727 | 79.7 | 5.72 |
| A0A0J9YUE9 | Dynamin-1 OS=Mus musculus GN=Dnm1 PE=1 SV=1 - [A0A0J9YUE9_MOUSE] | 20.29 | 11.74 | 18 | 6 | 7 | 7 | 835 | 94.0 | 6.64 |
| Q6PIE5 | Sodium/potassium-transporting ATPase subunit alpha-2 OS=Mus musculus GN=Atp1a2 PE=1 SV=1 - [AT1A2_MOUSE] | 56.35 | 20.78 | 7 | 5 | 17 | 20 | 1020 | 112.1 | 5.55 |
| Q8VDN2 | Sodium/potassium-transporting ATPase subunit alpha-1 OS=Mus musculus GN=Atp1a1 PE=1 SV=1 - [AT1A1_MOUSE] | 50.65 | 18.18 | 4 | 5 | 15 | 16 | 1023 | 112.9 | 5.45 |
| P11499 | Heat shock protein HSP 90-beta OS=Mus musculus GN=Hsp90ab1 PE=1 SV=3 - [HS90B_MOUSE] | 35.97 | 20.30 | 5 | 5 | 13 | 13 | 724 | 83.2 | 5.03 |
| P40142 | Transketolase OS=Mus musculus GN=Tkt PE=1 SV=1 - [TKT_MOUSE] | 27.85 | 16.85 | 1 | 5 | 8 | 9 | 623 | 67.6 | 7.50 |
| P58252 | Elongation factor 2 OS=Mus musculus GN=Eef2 PE=1 SV=2 - [EF2_MOUSE] | 26.52 | 10.61 | 5 | 5 | 7 | 9 | 858 | 95.3 | 6.83 |
| Q61316 | Heat shock 70 kDa protein 4 OS=Mus musculus GN=Hspa4 PE=1 SV=1 - [HSP74_MOUSE] | 22.10 | 12.96 | 2 | 5 | 8 | 8 | 841 | 94.1 | 5.24 |
| Q01853 | Transitional endoplasmic reticulum ATPase OS=Mus musculus GN=Vcp PE=1 SV=4 - [TERA_MOUSE] | 21.44 | 13.90 | 1 | 5 | 8 | 8 | 806 | 89.3 | 5.26 |
| Q8K2B3 | Succinate dehydrogenase [ubiquinone] flavoprotein subunit, mitochondrial OS=Mus musculus GN=Sdha PE=1 SV=1 - [SDHA_MOUSE] | 19.56 | 12.95 | 1 | 5 | 7 | 7 | 664 | 72.5 | 7.37 |

Immunoprecipitated proteins from a whole C57/BL6 mouse brain lysate using recombinant Fab 65 were electrophoretically separated onto a 12% polyacrylamaide gel. Protein bands were excised as they are numbered on Figure 4 and analyzed by mass spectrometry. As “Protein Band 10” is reffered the whole region indicated on the figure. The proteins listed, presended the highest counts regarding the parameter of unique peptides.
