# Supplementary material for: Application of antibody phage display to identify potential antigenic neural precursor cell proteins
Source: J Biol Res (Thessalon). 2020 Aug 2;27:14. doi: 10.1186/s40709-020-00123-4 (PMC7398072; doi:10.1186/s40709-020-00123-4)
Supplement: Supplementary file 3 — Additional file 3. PCR primers used in this study. Primers 1–19 were used for the amplification of IgG Fab fragments, while primers 20–21 for the sequencing of the isolated phagemids.Degenerative nucleotide symbols:K = G or T, S = C or G, M = A or C, W = A or T, R = A or G, Y = C or T. Restriction enzymes recognition sites are underlined, with A|CTAGT for SpeI, C|TCGAG for XhoI, T|CTAGA for XbaI and GAGCT|C for SacI. [file 40709_2020_123_MOESM3_ESM.docx]

**Additional file 3.**

**PCR primers used in this study.**

| 1 | 5’- SAK GTG CAG CTC GAG SAG TCA GGA CCT | 5’heavy chain Fd |
| --- | --- | --- |
| 2 | 5’- GAG GTY CAG CTC GAG CAR TCT GGA CCT | 5’heavy chain Fd |
| 3 | 5’- CAG GTC CAA CTC GAG CAG YCT GGG KCT | 5’heavy chain Fd |
| 4 | 5’- GAG GTT CAG CTC GAG CAG TCT GGR GCW G | 5’heavy chain Fd |
| 5 | 5’- GAR GTG AAG CTC GAG GAG WCT GGA SGA | 5’heavy chain Fd |
| 6 | 5’- GAG GTG AAG CTT CTC GAG TCT GGA GGT | 5’heavy chain Fd |
| 7 | 5’- GAA GTG MAG CTC GAG GAG TCT GGG GGA | 5’heavy chain Fd |
| 8 | 5’- AGG CTT ACT AGT ACA ATC CCT GGG CAC AAT | 3’ IgG1 |
| 9 | 5’- GTT CTG ACT AGT GGG CAC TCT GGG CTC | 3’ IgG2a |
| 10 | 5’- CTC CTT ACT AGT AGG ACA GGG GTT GAT TGT | 3’ IgG2b |
| 11 | 5’- GGG GGT ACT AGT CTT GGG TAT TCT AGG CTC | 3’ IgG3 |
| 12 | 5’- CCA GTT CCG AGC TCG TTG TGA CTC AGG AAT CT | 5’ κ light chain |
| 13 | 5’- CCA GTT CCG AGC TCG TGT TGA CGC AGC CGC CC | 5’ κ light chain |
| 14 | 5’- CCA GTT CCG AGC TCG TGC TCA CCC AGT CTC CA | 5’ κ light chain |
| 15 | 5’- CCA GTT CCG AGC TCC AGA TGA CCC AGT CTC CA | 5’ κ light chain |
| 16 | 5’- CCA GAT GTG AGC TCG TGA TGA CCC AGA CTC CA | 5’ κ light chain |
| 17 | 5’- CCA GAT GTG AGC TCG TCA TGA CCC AGT CTC CA | 5’ κ light chain |
| 18 | 5’- CCA GTT CCG AGC TCG TGA TGA CAC AGT CTC CA | 5’ κ light chain |
| 19 | 5’- GCG CCG TCT AGA ATT AAC ACT CAT TCC TGT TGA A | 3’ κ light chain |
| 20 | 5’- AAG ACA GCT ATC GCG ATT GCA G | sequencing |
| 21 | 5’- GCC CCC TTA TTA GCG TTT GCC ATC | sequencing |

Primers 1-19 were used for the amplification of IgG Fab fragments, while primers 20-21 for the sequencing of the isolated phagemids. Degenerative nucleotide symbols: K = G or T, S = C or G, M = A or C, W = A or T, R = A or G, Y = C or T. Restriction enzymes recognition sites are underlined, with A|CTAGT for *Spe*I, C|TCGAG for *Xho*I, T|CTAGA for *Xba*I and GAGCT|C for *Sac*I.
